# The Estrogen Receptor-β Expression in De Quervain’s Disease

**DOI:** 10.3390/ijms161125968

**Published:** 2015-11-04

**Authors:** Po-Chuan Shen, Ping-Hui Wang, Po-Ting Wu, Kuo-Chen Wu, Jeng-Long Hsieh, I-Ming Jou

**Affiliations:** 1 Department of Orthopedics, Tainan Hospital, Ministry of Health and Welfare, Tainan 70043, Taiwan; bcshen58@yahoo.com.tw; 2 Department of Orthopedics, Chi-Mei Medical Center, Tainan 71004, Taiwan; justin2051278@yahoo.com; 3 Department of Orthopedics, National Cheng Kung University College of Medicine, 1 Dashuei Road, Tainan 70101, Taiwan; anotherme500@gmail.com; 4 Department of Orthopedics, Kuo’s General Hospital, Tainan 70054, Taiwan; m004409@gmail.com; 5 Department of Nursing, Chung Hwa University of Medical Technology, 89 Wenhua 1st Street, Rende District, Tainan 71703, Taiwan

**Keywords:** estrogen receptor-β, de Quervain’s disease, inflammation, angiogenesis

## Abstract

Stenosing tenosynovitis of the first dorsal compartment of the wrist (a.k.a. de Quervain’s disease) is common but how estrogen is involved is still unknown. We previously reported that inflammation was involved in the pathogenesis of this ailment. In the present study, we extended our investigation of estrogen receptor (ER)-β expression to determine whether estrogen is involved in the pathogenesis of de Quervain’s. Intraoperative retinaculum samples were collected from 16 patients with the ailment. Specimens were histologically graded by collagen structure and immunohistochemically evaluated by quantifying the expression of ER-β, interleukin (IL)-1β and IL-6 (inflammatory cytokines), cyclooxygenase (COX)-2 (an inflammatory enzyme), and vascular endothelial growth factor (VEGF), and Von Willebrand’s factor (vWF). De Quervain’s occurs primarily in women. The female:male ratio in our study was 7:1. We found that ER-β expression in the retinaculum was positively correlated with disease grade and patient age. Additionally, disease severity was associated with inflammatory factors—IL-1β and IL-6, COX-2, and VEGF and vWF in tenosynovial tissue. The greater the levels of ER-β expression, tissue inflammation, and angiogenesis are, the more severe de Quervain’s disease is. ER-β might be a useful target for novel de Quervain’s disease therapy.

## 1. Introduction

De Quervain’s disease, “precisely defined as stenosing tenosynovitis of the first dorsal compartment” [1], is a common pathological condition of the wrist. Because of thickening of the sheath containing the abductor pollicis longus and extensor pollicis brevis tendons at the radial styloid process [2,3], it causes swelling and pain on the radial side of the wrist. Grip strength is gradually lost as the symptoms worsen. A number of non-surgical treatments, such as splinting, anti-inflammatory drugs (NSAIDs), and corticosteroid injections, are used to treat this disease [4,5]. Like trigger finger and carpal tunnel syndrome, de Quervain’s disease has been considered an overuse syndrome. They are all musculoskeletal disorders caused by repetitive hand posture and motion [6].

De Quervain’s disease is found more commonly in women during the later stages of pregnancy and in the early postpartum period [7,8]. Epidemiologic studies have shown that de Quervain’s disease occurs most often in women [7,8,9,10], in whom the prevalence rate is more than three times higher than in men in the working population [11]. Nevertheless, the etiology of the disease has not been clarified. Other studies have reported that carpal tunnel syndrome was highly associated with women who had undergone an oophorectomy or who were postmenopausal [12,13]. Among patients with carpal tunnel syndrome, estrogen receptors (ERs) are significantly expressed in the transverse carpal ligament and flexor tenosynovium. The expression of ERs in postmenopausal patients is related to their age [14,15]. The expression of ERs may be associated with female-dominant diseases.

Estrogen is a steroid hormone that affects the growth, differentiation, and development of female reproductive tissue [16]. The regulation of estrogen is essential for other key features of metabolism, such as food intake, body weight, glucose homeostasis, insulin sensitivity, body fat distribution, lipolysis and lipogenesis, inflammation, and cognition [17]. The biologically active form of estrogen, 17β-estradiol, is synthesized by the enzyme aromatase expressed in ovarian and peripheral tissue. Estrogen activity is mediated primarily by two nuclear receptors, abbreviated ER-α and ER-β. ER-α was first identified in healthy endometrium and in endometriosis [18]. The ER-β was then cloned from a rat prostate and a human testis; ER-β is specifically expressed in the testis, ovary, thymus, spleen, osteoblasts, fetus, and uterine endometrium [19,20]. They are both proteins with a high affinity for 17β-estradiol. In the absence of estrogen, ERs are sequestered within the nuclei of target cells and maintained in an inactive state. When estrogen is produced, it enters cells and binds to the ERs in estrogen-responsive cells. The steroid 17β-estradiol interacts with the ERs and acts as a transcription factor via direct DNA binding or binding to other docking transcription factors at basal promoter regions [16]. Together with ER-α, ER-β mediates many of the physiological effects of estrogens. The ER-β regulation is achieved by hormone binding and posttranslational modifications of the receptor.

We previously showed that the levels of several inflammatory factors, such as Mac387 (a macrophage marker), neutrophil elastase (a neutrophil marker), and cyclooxygenase-2 (COX-2) (also called prostaglandin-endoperoxide synthase [PTGS]), were all higher in patients with de Quervain’s disease [21]. The purpose of this study was to examine the expression of ERs and inflammatory factors in the tenosynovial tissue of patients with de Quervain’s disease and the involvement of estrogen in the pathogenesis of this disease. We showed that ER-β expression was higher in patients with de Quervain’s disease, that the degree of expression was related to the disease severity.

## 2. Results

### 2.1. The Expression of Estrogen Receptor (ER)-β Was Associated with the Severity of De Quervain’s Disease

Sixteen patients (14 women and 2 men; mean age: 55.62 years; range: 38–73 years) were recruited (Table 1). The mean age at menopause was 51.36 years (range: 49–56 years). None of the menstruating female patients were at gravidic or postpartum status. All patients had a clinical diagnosis of stenosing tenosynovitis (left: 10; right: 6), and all were completely symptom-free postoperatively with no triggering, recurrence, or volar subluxation of the abductor pollicis longus or extensor pollicis brevis tendons. Neither the disease duration nor the age at menopause determined the severity of the de Quervain’s disease. Samples of the retinaculum showed different disease severities in different parts from the first dorsal compartment of the wrist in the same patient.

**Table 1:** Demographic data and histological grading of patients with de Quervain’s disease.

Patient Number	Age at Surgery (Years)	Gender	Side	Duration (Months)	Corticosteroid Injection	Age at Menopause (Years)	Grade I	Grade II	Grade III
1	61	F	L	4	No	50	No	Yes	Yes
2	53	F	L	7	No	53	No	Yes	Yes
3	64	F	L	3	No	53	No	Yes	No
4	50	F	L	5	No	No	No	Yes	Yes
5	38	M	R	6	1	No	Yes	Yes	Yes
6	73	F	R	16	1	56	Yes	Yes	Yes
7	51	F	R	4	No	49	Yes	Yes	No
8	68	F	R	3	No	51	No	Yes	No
9	54	F	R	5	No	49	Yes	Yes	No
10	53	M	R	7	No	No	Yes	Yes	No
11	54	F	L	12	1	52	Yes	Yes	No
12	44	F	L	18	No	No	No	Yes	Yes
13	52	F	L	3	No	50	No	Yes	Yes
14	53	F	L	8	No	53	No	Yes	No
15	57	F	L	14	1	50	No	Yes	Yes
16	65	F	L	12	No	50	No	Yes	Yes

Mean age (years): 55.62 ± 8.87; Gender ratio (Female(F):Male(M)) = 7:1; Side (Left(L):Right(R)) = 10:6; Mean duration (months): 7.93 ± 4.91. Mean age at menopause (years): 51.36 ± 2.2.

**Figure 1:**
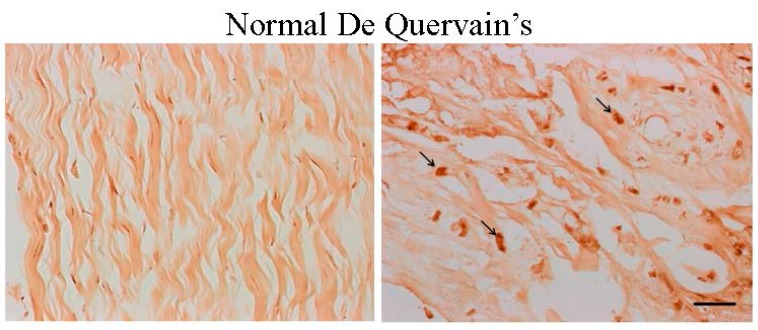
Compared with the samples from healthy controls, estrogen receptor (ER)-β-positive cells were visible in the nuclei of retinacular cells (arrows) in tissue samples from the representative slides of patients with grade II de Quervain’s disease (immunohistochemical staining, 400× magnification, scale bar = 10 μm).

**Figure 2:**
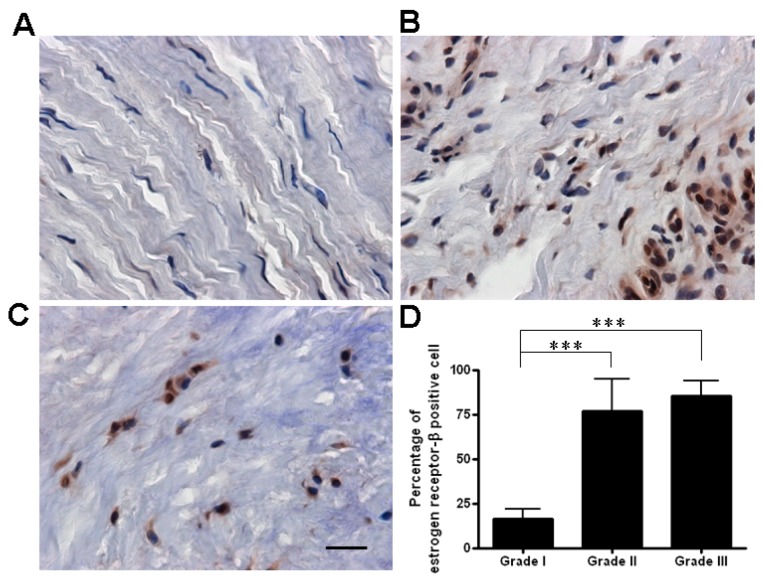
The percentage of ER-β-positive cells in grade I samples (**A**) was lower than in grade II (**B**) and grade III (**C**) samples (immunohistochemical staining, 400× magnification, scale bar = 10 μm); and (**D**) There were significantly more ER-β-positive cells in grade II and grade III areas than in grade I areas. The positive percentage was calculated as: (number of positive cells/total cells) × 100%. Data are mean ± standard deviation. *** *p* < 0.001.

The collagen structure in samples from normal controls was intact with a contiguous fiber bundle (Figure 1). Histopathologic analysis showed that the collagen structure was degraded: it had lost its parallel arrangement and had become more rounded in samples from patients with grade II de Quervain’s disease. Split and fragmented fibers were common and there was moderate cellular proliferation (Figure 1). In the grade I area samples, collagen fibers were slightly degraded: there was slight splitting between bundles (Figure 2A). The histochemical staining revealed that the expression of ER-β was localized in the nuclei in the retinacular cells. However, all retinacular cells were spindle-shaped and aligned in parallel. In the grade II area samples, there was moderately degraded collagen structure with some separation between bundles, increased waviness, and a loss of parallel alignment. The collagen fibers were moderately fragmented with intense cellular proliferation (Figure 2B). In the grade III area samples, the collagen fibers were severely degraded by a total loss of fiber orientation. Notably, the cell morphology had became more rounded and the cell viability had decreased (Figure 2C). The percentages of ER-β-positive cells in grade I, II, and III areas were 16.55% ± 5.29%, 76.99% ± 18.00% and 85.13% ± 8.71%, respectively (Figure 2D). The number of ER-β-positive cells in grades II and III, respectively, was significantly higher than that in grade I (*p* < 0.001). These results indicated that the expression of ER-β was related to the severity of de Quervain’s disease.

### 2.2. The Age Was Related to the Degree of ER-β Expression and Disease Severity

**Figure 3:**
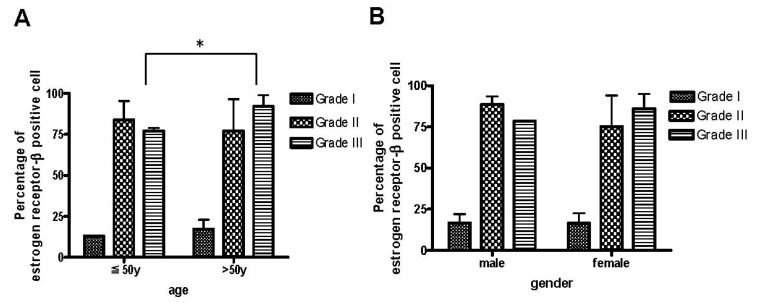
The percentage of ER-β-positive cells in the most severe tissue samples of de Quervain’s disease was age-dependent. (**A**) The expression of ER-β was higher in grade III area samples than in grade I and grade II area samples from patients >50 years old. The positive percentage was calculated as: (number of positive cells/total cells) × 100%. Data are mean ± standard deviation. * *p* < 0.05; and (**B**) No significant difference in the rate of expression of ER-β was found between men and women. Years(y): age.

We also analyzed factors related to the expression of ER-β. In grades I and II, there was no significant difference in the rate of expression of ER-β in the two different age groups. The expression of ER-β-positive cells was higher in grade III areas in patients >50 years old (92.03% ± 6.90%) than in those ≤50 (77.22% ± 1.69%) (*p* = 0.047) (Figure 3A). Our results suggested that age may affect the degree of ER-β expression and disease severity. Because only two of our 16 patients were men, there was no significant difference in the rate of expression of ER-β between men and women (Figure 3B).

### 2.3. Inflammation and Angiogenesis Occurred and Increased along with Disease Progression

**Figure 4:**
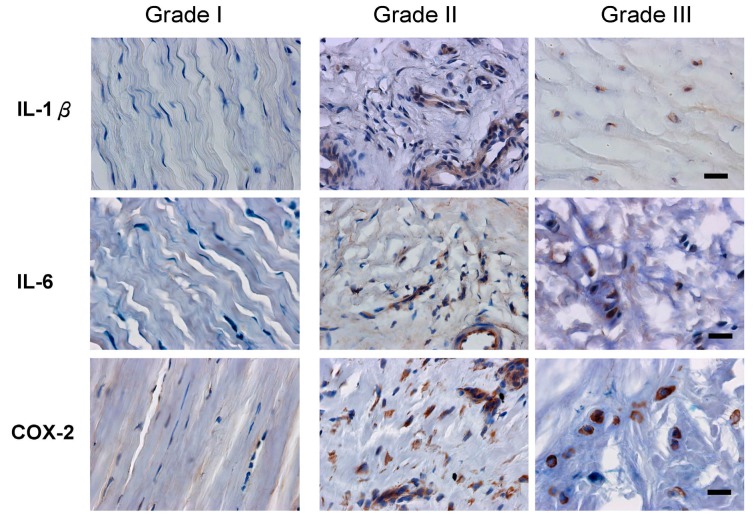
The expression of interleukin (IL)-1β, IL-6, and cyclooxygenase (COX)-2 in tissue was related to the disease severity. Serial sections of three grades (I-III) were stained with IL-1β, IL-6, and COX-2 antibodies, respectively. IL-1β, IL-6, and COX-2 were expressed in tissue from patients with grade II and grade III cases of de Quervain’s disease (immunohistochemical staining, 400× magnification, scale bar = 10 μm).

Immunochemical staining showed that the increased expression of three inflammatory factors (interleukin (IL)-1β, IL-6, and COX-2) was shown in grade II and III samples (Figure 4). The rates of expression of IL-1β and IL-6 in grade II samples (67.65% ± 16.17%, 67.23% ± 17.63%, respectively) and grade III samples (89.43% ± 8.53%, 84.16% ± 10.41%, respectively) were significantly higher than in grade I samples (13.08% ± 5.75%, 12.29% ± 8.72%, respectively) (Table 2). There were also significantly more COX-2-positive cells in grades II and III samples (87.07% ± 7.64%, 92.69% ± 5.51%, respectively) than in grade I samples (8.81% ± 6.87%).

**Figure 5:**
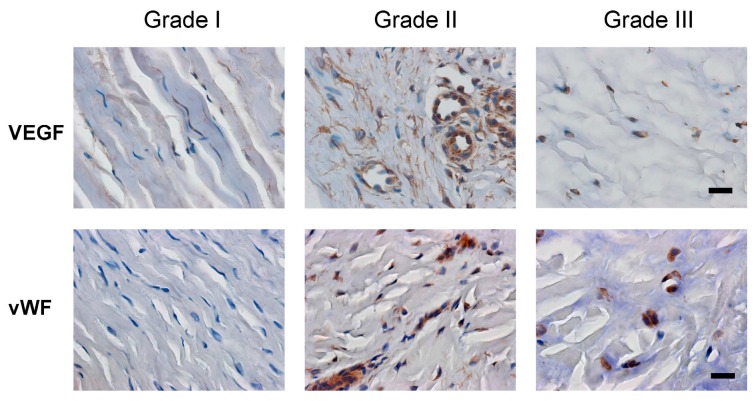
VEGF expression and vessel density in tissue samples of retinaculum were dependent upon disease severity. Serial sections of three grades (I–III) were stained with vascular endothelial growth factor (VEGF) and Von Willebrand’s factor (vWF) antibodies. More blood vessels were distributed in the grade II and grade III tissues of retinaculum (immunohistochemical staining, 400× magnification, scale bar = 10 μm).

Angiogenesis and inflammation are highly associated. Both of the angiogenesis-associated factors, vascular endothelial growth factor (VEGF) and Von Willebrand’s factor (vWF), were expressed in patients with grades II and III de Quervain’s disease (Figure 5). Similarly, the expression rate of VEGF and the number of vWF-positive vessels were associated with disease severity (Table 2). These results suggested that tissue inflammation and angiogenesis occurred and increased along with the progression of de Quervain’s disease.

**Table 2:** Comparison of the inflammatory and angiogenic factors change among three grades.

Factors	Grade I	Grade II	Grade III
IL-1β	13.08% ± 5.75%	67.65% ± 16.17% ***	89.43% ± 8.53% ***
IL-6	12.29% ± 8.72%	67.23% ± 17.63% ***	84.16% ± 10.41% ***
COX-2	8.81% ± 6.87%	92.69% ± 5.51% ***	87.07% ± 7.64% ***
VEGF	12.33% ± 4.60%	75.78% ± 17.70% ***	90.63% ± 4.45% ***
vWF	5.96% ± 0.10%	48.99% ± 23.15%	70.21% ± 15.54% *

IL, interleukin; COX, cyclooxygenase; VEGF, vascular endothelial growth factor; vWF, von Willebrand’s factor. Data are a percentage of positively stained cells, expressed as the mean ± standard deviation, and analyzed using one-way analysis of variance (ANOVA) and Bonferroni’s multiple comparison test (* *p* < 0.05, *** *p* < 0.001 compared with the grade I group).

## 3. Discussion

We found that ER-β was expressed in the tenosynovial tissue of patients with de Quervain’s disease. The expression levels were related to disease severity. ER-β is highly regulated by estrogen [14,15]. Interestingly, ER-β is upregulated in the tenosynovial tissue of postmenopausal woman with idiopathic carpal tunnel syndrome, the pathogenesis of which has been associated with altered expression of ERs [15]. It may also occur in de Quervain’s disease. The pathological change of de Quervain’s disease is primarily an extensor retinaculum thickened by fibrosis and fibrocartilaginous metaplasia. The thickening of the fibrous tendon sheath, especially in the central part, showed greater proliferation of macrophages and more angiogenesis [22]. We found that ER-β expression started when de Quervain’s disease began, and that it peaked during the moderate stage: there was no significant difference in ER-β expression during more severe stages. It is very likely that estrogen aggravated inflammation and angiogenesis through the increased ER-β expression. Of note, immunohistochemical staining revealed no significant difference in ER-α expression between patients and normal controls. Nevertheless, a more sensitive method, real-time polymerase chain reaction (RT-PCR), was used [23]. The ratio of ER-α to ER-β changed in myometrium of women during the transition of menopause. In postmenopausal myometrium, ER-β expression was significantly higher than it was in premenopausal myometrium, but the level of ER-α mRNA was relatively lower. A significant change in the ratio of ER-β to ER-α expression is also seen in human endometriosis and metastatic lesions of uterine endometrial cancers [24,25]. ER-β expression can be regulated by DNA methylation of the ER-β promoter region [26]. Deficient methylation of the ER-β promoter results in pathological overexpression of ER-β in endometriotic stromal cells [27,28]. High ER-β expression is considered a dominant-negative regulator of ER-α that modulates its transcriptional responses to estrogens [29]. ER-β overexpression in these cells reduced ER-α mRNA and protein levels. This might also occur in de Quervain’s disease. A severely high ER-β-to-ER-α ratio could be pathogenic in estrogen-responding tissue. After Raw 264.7 cells had been treated with estrogen, the expression of ER-β and receptor activator of nuclear factor-κβ (RANK) both increased [30], which suggested that estrogen signaling is important for modulating inflammation in diseases. Therefore, ER-β expression is believed to be a target in patients treated with adjuvant tamoxifen, which has an anti-estrogenic effect in breast and lung cancers [31,32]. Because of the success of breast cancer treatments with drugs that act on the ER-β, it was natural to think of ER-β as a potential treatment target for cancers and certain diseases. Three selective estrogen receptor modulators, raloxifene, toremifene and tamoxifen have mixed agonist and antagonist activity, depending on the target tissue [33]. They act on both ER-α and ER-β. Tamoxifen can have serious side effects. Therefore, drugs selective for ER-β could theoretically ameliorate the symptoms without having the side effects linked to drugs traditionally associated with ER-α. To this end, a selective antagonist of ER-β, 4-[2-Phenyl-5,7-bis(trifluoromethyl) pyrazolo[1,5-a]pyrimidin-3-yl] phenol (PHTPP) had been shown to decrease the mucus plugging in chronic inflammatory conditions of the airway and reduce bladder cancer cell growth and invasion [34,35]. PHTPP might be considered as a therapeutic alternative for de Quervain’s disease in post-menopausal women.

In addition to reporting on ER-β immunoreactivities in tenosynovial tissue, we recently detected both neutrophils and macrophages in the tissue of patients, which suggested that inflammation is involved in the progression of de Quervain’s disease [21]. It is likely that ER-β stimulates prostaglandin production in tenosynovial tissue by inducing COX-2 expression, which contributes to inflammation. Like the pathogenesis of osteoarthritis [36,37], macrophages that have infiltrated the synovial membrane produce angiogenic factors like VEGF and inflammatory cytokines like IL-1, IL-6, and COX-2, which further impair the homeostasis of the tendons. Chronic inflammation and angiogenesis might be the primary causes of fibrosis-thickened extensor retinaculum. Studies of athletic injuries have demonstrated that sex hormone fluctuations could change the composition of the ligament and predispose it to the higher injury rate in women [38,39]. A recent case report [40] said that bilateral de Quervain’s disease was probably related to aromatase inhibitors (AIs), which reduce estrogen levels [40,41]. A fluctuation in estrogen level might cause the aberrant expression of ER-β and the inflammation that results in de Quervain’s tenosynovitis. How changes in estrogen levels affect changes in ER-β expression and subsequent biological responses in the microenvironment of the wrist requires additional investigation.

Females are more likely to develop autoimmune and inflammatory diseases because of sexual dimorphism [42,43]. For instance, the incidence of multiple sclerosis is 3–5 times higher in women. However, how estrogen modulates inflammation is still not clear. ERs and their ligands have long been known to be anti-inflammatory. Saijo *et al.* [44] reported that an ER-specific ligand, 5-androsten-3β,17β-diol (ADIOL), endogenously controlled and potently inhibited the transcriptional activation of inflammatory responses through the mechanism of ER-β. ADIOL is anti-inflammatory, and its expression is controlled by inducers and suppressors of inflammation. Therefore, in a proinflammatory environment, a reduction in ADIOL can block an ER-β-dependent anti-inflammatory pathway driven by the local production of endogenous ligands. Interestingly, 17β-estradiol itself is not potently anti-inflammatory. Saijo *et al.* [44] found that increasing 17β-estradiol levels reduced ADIOL’s anti-inflammatory effect. ADIOL has a potent anti-inflammatory effect in men because men have relatively low levels of 17β-estradiol. These findings may partially explain why men have much lower incidences of this type of disease than do women.

Of our sixteen patients, nine had symptoms that were more severe and reached grade III of de Quervain’s disease. Thirteen of these patients were >50 years old, and three were ≤50. The percentage of ER-positive cells in the samples of grade III areas from the older group was significantly higher than in the samples of grade III areas from the younger group, which indicated that age might be a factor that determines the expression levels of ER-β and the severity of de Quervain’s disease. Women entering the peri-menopausal phase were between 40 and 45 years old; the worldwide average age at menopause is approximately 51 years [45], which corresponds to the age in our study (51.36 years old). The abrupt changes in estrogen expression during this menopause phase might explain why de Quervain’s disease develops.

The major limitation of this study is that only two of our patients with de Quervain’s disease were men, which precluded the possibility of finding a significant gender-based difference in ER-β expression and disease severity. Moreover, the sparseness of samples from persons less than 50 years old made it difficult to analyze if there was a dose-response in groups with different grades. However, one of the men did have high levels of ER-β expression and, perhaps as a consequence, a more severe case of de Quervain’s disease. The development of de Quervain’s tenosynovitis was thought to be caused by prolonged and repeated thumb extension combined with non-neutral wrist postures [2]. A continuous mechanical stress might exacerbate an inflammatory reaction. Even in men, a proinflammatory environment might activate steroid-metabolizing enzymes and trigger the estrogen signaling. In both genders, the fluctuation of estrogen combined with overused tissue, it was hypothesized, was probably involved in the disease. The way they mutually affect each other in the pathogenesis of de Quervain’s disease, therefore, requires further investigation. Due to the lack of normal healthy tissues, we used the fresh cadavers as the control group. They were all organ donors. Before donation, detailed examinations were performed. Based on the report of physical examination and inquiry of medical history, no de Quervain complaints were noted in these controls.

## 4. Experimental Section

Following Institutional Review Board approval, we enrolled and analyzed 16 patients who had been diagnosed with de Quervain’s disease and underwent surgery between 9 January and 4 December 2014, in the Orthopedics Department at our hospital. Healthy tissue samples of retinaculum for the control group were obtained from three fresh cadavers. The specimens from patients and controls were histologically and immunohistochemically compared. This prognostic study investigates the pathogenesis of de Quervain’s disease (Level II of Evidence). Patients who had previous trauma or surgery on the dorsal compartments of their wrists and patients with systemic inflammatory disorders were not included in the study. Ethical approval was provided by the Institutional Review Board of National Cheng-Kung University Hospital in 20 December, 2013 (IRB number: ER-102-241).

### 4.1. The Disease Diagnosis

The diagnosis of de Quervain’s disease was made based on medical history and the findings of clinical examinations of pain with swelling or nodule formation over the thumb side of the wrist. The indications of active tenosynovitis were including tenderness over the side of the wrist at the base of the thumb, pain along the extensor and abductor pollicis tendons, pain with a passive stretch, and locking. At the initial presentation, the treating physicians collected detailed demographic data (age, gender, body mass index, hand dominance, the digits involved at presentation, and the involvement of other hand disorders), and they addressed the disease duration and severity of the symptoms, including the treatment history on local drug injection. For those patients who had been included, the medical history of steroid injection needed to be longer than three months. For those patients who had a recent history of steroid injection (<three months) had been excluded. The indication for surgical release was considered when disease duration was longer than three months, and unsuccessful treatment with the conservative treatments of splinting, physical therapy, NSAIDs, or steroid injections.

### 4.2. Tissue Collection and Section Evaluation

At the time of surgical decompression, a biopsy of the dorsal portion of the extensor retinaculum and the adjacent fibrous sheath and synovium tissue superficial to the tendons was done longitudinally. The control tissues were obtained from fresh cadavers, two female (48 and 64 years old, respectively) and one male (71 years old). All specimens were fixed in formalin, dehydrated in 30% sucrose and cut into 10-μm-thick cryosections for hematoxylin and eosin (H & E) staining and immunohistochemical staining. Slides of the different stainings were scanned and digital images taken. The severity of the disease was graded using a collagen structure grading method previously described [21,46,47]. Briefly, based on the collagen structure of the extracelluar matrix, all samples were categorized as grade I (collagen structure with slight waviness and minimal splitting between intact fiber bundles was slightly degraded), grade II (collagen structure with moderate waviness, fiber fragmentation, some separation between fiber bundles and loss of parallel arrangement was moderately degraded), or grade III collagen structure with total loss of fiber orientation and severe fragmentation of fibers was severely degraded). The samples were separately graded by two physicians who worked independently. Their results were compared and a third physician cast a vote when the first two grades were not identical.

### 4.3. Immunohistochemical Staining

Serial sections were first treated with 0.03% peroxide for 10 min to quench endogenous peroxidase. The sections were rinsed for 20 min with blocking and permeabilizing solution that contained 5% normal goat serum, 0.1% bovine serum albumin, and 0.2% Triton, and then incubated at 4 °C overnight with each of the following primary antibodies: rabbit anti-estrogen receptor-β (Abcam, Cambridge, MA, USA), rabbit anti-IL-1β (OriGene Technologies, Rockville, MD, USA), rabbit anti-IL-6 (Abcam), rabbit anti-COX-2 (Abcam), mouse anti-VEGF monoclonal antibody (VG-1; Abcam), and rabbit anti-vWF (Proteintech, Chicago, IL, USA). After they had been sequentially incubated with the appropriate secondary antibody (1:400) (Jackson ImmunoResearch Laboratories, West Grove, PA, USA) for 2 h at room temperature with diaminobenzidine (DAB) as the substrate chromogen (BSB0205; Bio SB, Santa Barbara, CA, USA), the sections were counterstained with hematoxylin.

### 4.4. Cell Counting

Sections were examined under a Zeiss light microscope (Jena, Germany). The immunoreactivity of ER-β, IL-1β, IL-6, COX-2, VEGF, and vWF were evaluated. Five fields of each section (randomly selected from each grade) were examined at 400× magnification. It was possible for the samples to show different grades in the same patient. Using an image processing program ImageJ [48], the percentage of positive cells and the total cell population in each chosen field was counted. The positive percentage was calculated as: (number of positive cells/total cells) × 100%.

### 4.5. Statistical Analysis

All values are mean ± standard deviation. GraphPad Prism (La Jolla, CA, USA) software was used to do one-way ANOVA and Bonferroni’s multiple comparison tests. Significance was set at *p* ≤ 0.05.

## 5. Conclusions

We have shown that ER-β expression was higher in patients with de Quervain’s disease, that the degree of expression was related to the disease severity, and that the disease severity was also associated with tissue inflammation and angiogenesis. In addition to a ligand, the level of ER-β might determine how retinaculum tissue responds to it. The possible effects of ER-β for treating cancer have been documented; however, to the best of our knowledge, this is the first study to show that ER-β and de Quervain’s disease are associated. ER-β might be a useful target for treating de Quervain’s disease.

## References

[1] De Quervain F. (1997). On a form of chronic tendovaginitis by Dr. Fritz de Quervain in la Chaux-de-Fonds. 1895. Am. J. Orthop..

[2] Moore J.S. (1997). De Quervain’s tenosynovitis. Stenosing tenosynovitis of the first dorsal compartment. J. Occup. Environ. Med..

[3] Ilyas A.M., Ast M., Schaffer A.A., Thoder J. (2007). De quervain tenosynovitis of the wrist. J. Am. Acad. Orthop. Surg..

[4] Peters-Veluthamaningal C., van der Windt D.A., Winters J.C., Meyboom-de Jong B. (2009). Corticosteroid injection for de Quervain’s tenosynovitis. Cochrane Database Syst. Rev..

[5] Di Sante L., Martino M., Manganiello I., Tognolo L., Santilli V. (2013). Ultrasound-guided corticosteroid injection for the treatment of de Quervain’s tenosynovitis. Am. J. Phys. Med. Rehabil..

[6] Laoopugsin N., Laoopugsin S. (2012). The study of work behaviours and risks for occupational overuse syndrome. Hand Surg..

[7] Read H.S., Hooper G., Davie R. (2000). Histological appearances in post-partum de Quervain’s disease. J. Hand Surg. Br..

[8] Johnson C.A. (1991). Occurrence of de Quervain’s disease in postpartum women. J. Fam. Pract..

[9] (2010). Tendon Trouble in the Hands: De Quervain’s Tenosynovitis and Trigger Finger. Women are more likely than men to develop these painful conditions. Harv. Women Health Watch.

[10] Wolf J.M., Sturdivant R.X., Owens B.D. (2009). Incidence of de Quervain’s tenosynovitis in a young, active population. J. Hand Surg. Am..

[11] Petit Le Manac’h A., Roquelaure Y., Ha C., Bodin J., Meyer G., Bigot F., Veaudor M., Descatha A., Goldberg M., Imbernon E. (2011). Risk factors for de Quervain’s disease in a French working population. Scand. J. Work Environ. Health.

[12] Pascual E., Giner V., Arostegui A., Conill J., Ruiz M.T., Pico A. (1991). Higher incidence of carpal tunnel syndrome in oophorectomized women. Br. J. Rheumatol..

[13] Hakim A.J., Cherkas L., el Zayat S., MacGregor A.J., Spector T.D. (2002). The genetic contribution to carpal tunnel syndrome in women: A twin study. Arthritis Rheum..

[14] Toesca A., Pagnotta A., Zumbo A., Sadun R. (2008). Estrogen and progesterone receptors in carpal tunnel syndrome. Cell Biol. Int..

[15] Kim J.K., Hann H.J., Kim M.J., Kim J.S. (2010). The expression of estrogen receptors in the tenosynovium of postmenopausal women with idiopathic carpal tunnel syndrome. J. Orthop. Res..

[16] Pavao M., Traish A.M. (2001). Estrogen receptor antibodies: Specificity and utility in detection, localization and analyses of estrogen receptor α and β. Steroids.

[17] Clegg D.J. (2012). Minireview: The year in review of estrogen regulation of metabolism. Mol. Endocrinol..

[18] Green S., Walter P., Kumar V., Krust A., Bornert J.M., Argos P., Chambon P. (1986). Human oestrogen receptor cDNA: Sequence, expression and homology to v-*erb*-*A*. Nature.

[19] Kuiper G.G., Enmark E., Pelto-Huikko M., Nilsson S., Gustafsson J.A. (1996). Cloning of a novel receptor expressed in rat prostate and ovary. Proc. Natl. Acad. Sci. USA.

[20] Mosselman S., Polman J., Dijkema R. (1996). ERβ: Identification and characterization of a novel human estrogen receptor. FEBS Lett..

[21] Kuo Y.L., Hsu C.C., Kuo L.C., Wu P.T., Shao C.J., Wu K.C., Wu T.T., Jou I.M. (2015). Inflammation is present in de Quervain disease-correlation study between biochemical and histopathological evaluation. Ann. Plast. Surg..

[22] Clarke M.T., Lyall H.A., Grant J.W., Matthewson M.H. (1998). The histopathology of de Quervain’s disease. J. Hand Surg. Br..

[23] Sakaguchi H., Fujimoto J., Aoki I., Tamaya T. (2003). Expression of estrogen receptor α and β in myometrium of premenopausal and postmenopausal women. Steroids.

[24] Brandenberger A.W., Lebovic D.I., Tee M.K., Ryan I.P., Tseng J.F., Jaffe R.B., Taylor R.N. (1999). Oestrogen receptor (ER)-α and ER-β isoforms in normal endometrial and endometriosis-derived stromal cells. Mol. Hum. Reprod..

[25] Fujimoto J., Sakaguchi H., Aoki I., Toyoki H., Tamaya T. (2002). Clinical implications of the expression of estrogen receptor-α and -β in primary and metastatic lesions of uterine endometrial cancers. Oncology.

[26] Swedenborg E., Power K.A., Cai W., Pongratz I., Rüegg J. (2009). Regulation of estrogen receptor β activity and implications in health and disease. Cell. Mol. Life Sci..

[27] Bulun S.E., Monsavais D., Pavone M.E., Dyson M., Xue Q., Attar E., Tokunaga H., Su E.J. (2012). Role of estrogen receptor-β in endometriosis. Semin. Reprod. Med..

[28] Xue Q., Lin Z., Cheng Y.H., Huang C.C., Marsh E., Yin P., Milad M.P., Confino E., Reierstad S., Innes J. (2007). Promoter methylation regulates estrogen receptor 2 in human endometrium and endometriosis. Biol. Reprod..

[29] Trukhacheva E., Lin Z., Reierstad S., Cheng Y.H., Milad M., Bulun S.E. (2009). Estrogen receptor (ER) β regulates ERα expression in stromal cells derived from ovarian endometriosis. J. Clin. Endocrinol. Metab..

[30] Galal N., El-Beialy W.R., Deyama Y., Yoshimura Y., Suzuki K., Totsuka Y. (2007). Novel effect of estrogen on RANK and c-fms expression in RAW 264.7 cells. Int. J. Mol. Med..

[31] Younes M., Honma N. (2011). Estrogen receptor β. Arch. Pathol. Lab. Med..

[32] Monica V., Longo M., Felice B., Scagliotti G.V., Papotti M., Novello S. (2012). Role of hormone receptor expression in patients with advanced-stage lung cancer treated with chemotherapy. Clin. Lung Cancer.

[33] Jia M., Dahlman-Wright K., Gustafsson J.Å. (2015). Estrogen receptor α and β in health and disease. Best Pract. Res. Clin. Endocrinol. Metab..

[34] Tam A., Wadsworth S., Dorscheid D., Man S.F., Sin D.D. (2014). Estradiol increases mucus synthesis in bronchial epithelial cells. PLoS ONE.

[35] Hsu I., Chuang K.L., Slavin S., Da J., Lim W.X., Pang S.T., O’Brien J.H., Yeh S. (2014). Suppression of ERβ signaling via ERβ knockout or antagonist protects against bladder cancer development. Carcinogenesis.

[36] Shen P.C., Lu C.S., Shiau A.L., Lee C.H., Jou I.M., Hsieh J.L. (2013). Lentiviral small hairpin RNA knockdown of macrophage inflammatory protein-1γ ameliorates experimentally induced osteoarthritis in mice. Hum. Gene Ther..

[37] Hsieh J.L., Shen P.C., Shiau A.L., Jou I.M., Lee C.H., Wang C.R., Teo M.L., Wu C.L. (2010). Intraarticular gene transfer of thrombospondin-1 suppresses the disease progression of experimental osteoarthritis. J. Orthop. Res..

[38] Liu S.H., Al-Shaikh R.A., Panossian V., Finerman G.A., Lane J.M. (1997). Estrogen affects the cellular metabolism of the anterior cruciate ligament. A potential explanation for female athletic injury. Am. J. Sports Med..

[39] Yu W.D., Panossian V., Hatch J.D., Liu S.H., Finerman G.A. (2001). Combined effects of estrogen and progesterone on the anterior cruciate ligament. Clin. Orthop. Relat. Res..

[40] Papadimitriou K., Kountourakis P., Morakis E., Vassiliou V., Barbounis V., Ardavanis A. (2012). Bilateral de Quervain syndrome after aromatase inhibitor administration: A case report and review of the literature. Case Rep. Med..

[41] Pavone M.E., Bulun S.E. (2013). Clinical review: The use of aromatase inhibitors for ovulation induction and superovulation. J. Clin. Endocrinol. Metab..

[42] Bearoff F., Case L.K., Krementsov D.N., Wall E.H., Saligrama N., Blankenhorn E.P., Teuscher C. (2015). Identification of genetic determinants of the sexual dimorphism in CNS autoimmunity. PLoS ONE.

[43] Kovacs W.J., Olsen N.J. (2011). Sexual dimorphism of RA manifestations: Genes, hormones and behavior. Nat. Rev. Rheumatol..

[44] Saijo K., Collier J.G., Li A.C., Katzenellenbogen J.A., Glass C.K. (2011). An ADIOL-ERβ-CtBP transrepression pathway negatively regulates microglia-mediated inflammation. Cell.

[45] Kok H.S., van Asselt K.M., van der Schouw Y.T., Peeters P.H., Wijmenga C. (2005). Genetic studies to identify genes underlying menopausal age. Hum. Reprod. Update.

[46] Movin T., Gad A., Reinholt F.P., Rolf C. (1997). Tendon pathology in long-standing achillodynia. Biopsy findings in 40 patients. Acta Orthop. Scand..

[47] Chen J., Wang A., Xu J., Zheng M. (2010). In chronic lateral epicondylitis, apoptosis and autophagic cell death occur in the extensor carpi radialis brevis tendon. J. Shoulder Elbow Surg..

[48] ImageJ Image Processing and analysis in Java. http://imagej.nih.gov/ij/.

